# Proteomic Profiling of Cytosolic Glutathione Transferases from Three Bivalve Species: *Corbicula fluminea*, *Mytilus galloprovincialis* and *Anodonta cygnea*

**DOI:** 10.3390/ijms15021887

**Published:** 2014-01-27

**Authors:** José Carlos Martins, Alexandre Campos, Hugo Osório, Rute da Fonseca, Vítor Vasconcelos

**Affiliations:** 1 CIIMAR/CIMAR—Interdisciplinary Centre of Marine and Environmental Research, University of Porto, Rua dos Bragas 289, 4050-123 Porto, Portugal; E-Mails: acampos@ciimar.up.pt (A.C.); rute.r.da.fonseca@gmail.com (R.F.); vmvascon@fc.up.pt (V.V.); 2 IPATIMUP—Institute of Molecular Pathology and Immunology of the University of Porto, 4200-465 Porto, Portugal; E-Mail: hosorio@ipatimup.pt; 3 Faculty of Medicine, University of Porto, 4200-319 Porto, Portugal; 4 Centre for GeoGenetics, Natural History Museum of Denmark, University of Copenhagen, Øster Voldgade 5-7, 1350 Copenhagen, Denmark; 5 Department of Biology, Faculty of Sciences, Porto University, Rua do Campo Alegre, 4069-007 Porto, Portugal

**Keywords:** glutathione transferases, *Corbicula fluminea*, *Anodonta cygnea*, *Mytilus galloprovincialis*, detoxification, proteomics

## Abstract

Suspension-feeding bivalves are considered efficient toxin vectors with a relative insensitivity to toxicants compared to other aquatic organisms. This fact highlights the potential role of detoxification enzymes, such as glutathione transferases (GSTs), in this bivalve resistance. Nevertheless, the GST system has not been extensively described in these organisms. In the present study, cytosolic GSTs isoforms (cGST) were surveyed in three bivalves with different habitats and life strategies: *Corbicula fluminea*, *Anodonta cygnea* and *Mytilus galloprovincialis*. GSTs were purified by glutathione-agarose affinity chromatography, and the collection of expressed cGST classes of each bivalve were identified using a proteomic approach. All the purified extracts were also characterized kinetically. Results reveal variations in cGST subunits collection (diversity and properties) between the three tested bivalves. Using proteomics, four pi-class and two sigma-class GST subunits were identified in *M. galloprovincialis. C. fluminea* also yielded four pi-class and one sigma-class GST subunits. For *A. cygnea*, two mu-class and one pi-class GST subunits were identified, these being the first record of GSTs from these freshwater mussels. The affinity purified extracts also show differences regarding enzymatic behavior among species. The variations found in cGST collection and kinetics might justify diverse selective advantages for each bivalve organism.

## 1. Introduction

Aquatic organisms are recurrently exposed to numerous environmental contaminants. Bivalves, as sessile filter-feeding organisms, are one of the most threatened organisms by these environmental stressors. To avoid or reduce potential toxic insult, these mollusks present behavioral and physiological mechanisms, which may differ among species. Glutathione transferases (GSTs) play a significant role in cellular defense against chemically-induced toxicity. Drug-metabolizing enzymes belonging to the GST superfamily are multifunctional phase II proteins primarily involved in the cellular detoxification of both endogenous and exogenous compounds [1]. The GST defensive system acts mainly by catalyzing the conjugation of the thiol group of reduced glutathione (GSH) with the electrophilic sites of noxious compounds [2]. This represents the first step of the mercapturic acid detoxification pathway, resulting in more water soluble peptide derivatives than the parent toxin [3]. GSTs are also involved in intracellular transport, the biosynthesis of hormones and protection against oxidative stress [1,3,4]. GSTs are ubiquitous enzymes found from bacteria to humans. Three major families have been identified: the cytosolic, the mitochondrial and the microsomal GSTs [3]. Cytosolic GSTs (cGST) are by far the most abundant GST family, divided into several classes. In mammals, cGSTs are grouped in alpha, mu, pi, sigma, omega, zeta and theta classes [3]. Additional classes are specific to non-mammalian organisms and include phi (plants), tau (plants), rho (fish, bivalves), delta (insects), epsilon (insects) and beta (bacteria) GSTs [5,6]. The number of isoforms per class varies widely, ranging from one to forty [1].

GSTs are a relevant part of the adaptive response mechanisms to chemical stress. Thus, these detoxification enzymes have been used as biomarkers for the monitoring of pollution in aquatic systems. In fact, several studies have already demonstrated a correlation between the presence of xenobiotics and the activity and expression of various isoforms of GSTs in bivalves [7–11]. Better characterization of the GSTs of bivalve mollusks will be of value to the fields of toxicology and environmental quality assessment. Compared with vertebrates, there is little information available concerning GSTs from invertebrate aquatic organisms, such as mollusks. Surprisingly, GST isoforms started to be isolated and characterized from mussels more than two decades ago [12]. Several different classes of bivalve GSTs have been identified and reported in GenBank (Table 1), although only a few have been cloned and characterized. Isolation and characterization of particular types of GSTs have been studied from bivalve species, such as *M. edulis* [12–14], *M. galloprovincialis* [12,15,16], *Perna perna* [16], *C. fluminea* [17,18], *Asaphis dichotoma* [19], *Mercenaria mercenaria* [20], *Ruditapes decussatus* [21], *Ruditapes philippinarum* [22,23], *Atactodea striata* [24] and *Chlamys islandica* [25].

In this study, we use a proteomic approach involving affinity chromatography coupled with mass spectrometry to provide a detailed assessment of GST protein diversity in *C. fluminea*, *A. cygnea* and *M. galloprovincialis*. We aimed to identify cGST isoforms by two-dimensional gel electrophoresis (2DE) and to characterize cGST activity and kinetics in the three bivalves. The profiling by two-dimensional gel electrophoresis gives both an idea of the number of subunits evident in each species and an indication of their relative abundance. The comparison of GSTs proteomes and kinetic data provides an important perspective on GST diversity and potential in the cellular defense to xenobiotics exposure of bivalves with different habitats and life strategies.

## 2. Results and Discussion

GSTs represent a superfamily of ubiquitous enzymes with many physiological functions, including detoxification [1,3]. They have well-established roles in protecting cells against the products of oxidative damage or the metabolism of xenobiotics. In fact, the number of GST isoforms and their catalytic promiscuity might act as a fitness advantage in organisms subjected to environmental chemical stress situations. An excellent example is the resistance of insects to xenobiotics (organophosphate, organochlorines and pyrethroids), which typically involves increases in the metabolic capabilities of detoxificative enzymes and where GSTs have a major role [26]. However, it is difficult to attribute particular functions to any individual or group of GSTs, since, in many contexts, they act as a complex. In this way, the knowledge of the diversity of GSTs expressed can be explored in order to identify the specific isoforms involved, individually or not, in xenobiotic transformation and degradation. The current work selectively examines the expression of the members of the GST superfamily in three different bivalve mollusks with diverse life strategies and habitats. Marine mussels, such as *M. galloprovincialis*, are suspension feeders commonly living in dense masses at the intertidal and subtidal level of coastal waters and estuaries [27]. They are often dominant in their habitats, probably due to their remarkable plasticity regarding environmental changes, and they are extensively used as bioindicators of chemical water pollution [28]. The Asian freshwater clam, *C. fluminea*, is an invasive species of great success, due to its ecological plasticity and reproductive capacity, with potentially negative effects on the conservation of native species of freshwater bivalves [29]. This benthic species is a suspension feeder that can be found in rivers, canals, reservoirs and lakes [29]. In contrast, *A. cygnea* is a lacustrine species with a very restricted distribution, found only in large, but shallow, ponds [30].

### 2.1. 2DE cGSTs Identification

**Figure 1.**
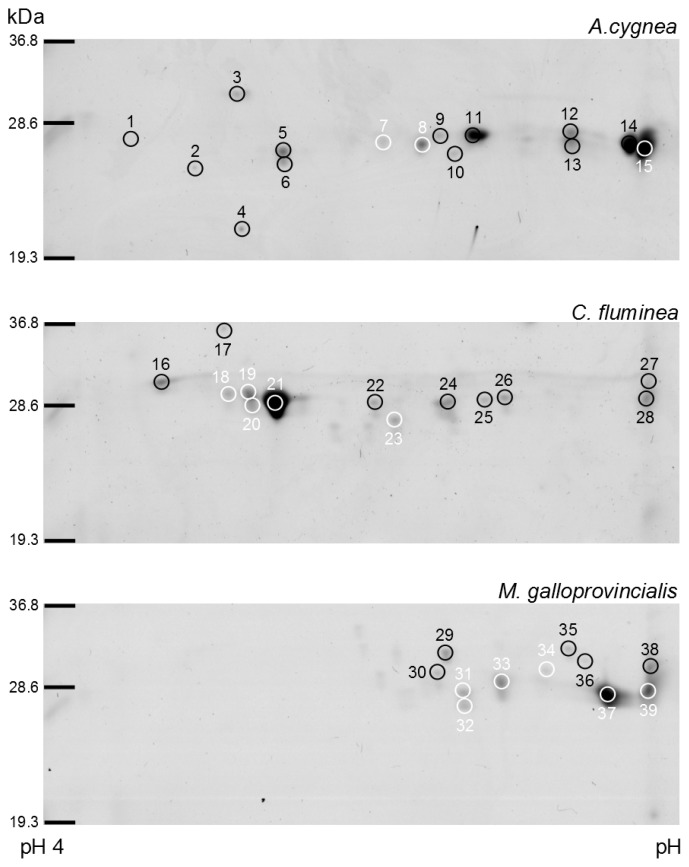
Protein expression profiles in two-dimensional gel electrophoresis (2DE) gels obtained from the affinity chromatography purified cGST of *A. cygnea*, *C. fluminea* and *M. galloprovincialis* (the numbers in white represent the identified cGSTs).

The affinity-purified cGST fractions from *A. cygnea*, *C. fluminea* and *M. galloprovincialis* were analyzed by 2DE followed by protein identification using mass spectrometry procedures. Soluble GSTs are, as a general rule, biologically active as dimers of subunits of 23–30 kDa [1]. Therefore, preferential attention was paid to the low molecular weight proteins detected on 2DE gels (Figure 1). In this range, 15, 13 and 11 protein spots per gel were visualized for *A. cygnea*, *C. fluminea* and *M. galloprovincialis*, respectively, using Coomassie staining and PDQuest 2D Analysis Software. These protein spots were excised and analyzed by MALDI-TOF-MS/MS (Table 2). The results reveal a discrepancy between the number of spots visualized on the gels and the identified ones. Of the 15 spots for *A. cygnea*, three were identified as cGSTs subunits, with molecular masses between 27.4 and 28.0 kDa and isoelectric points (pI) values between 5.5 and 6.7. Spots 7 and 8 were identified as putative mu-class GST subunits (B3VDE4 and A7LFK1) and spot 15 as a putative pi-class GST subunit (E1B2Z8). For *C. fluminea*, five of the 13 spots were identified as cGSTs subunits, with molecular masses between 28.2 and 30.7 kDa and pI values between 4.8 and 5.6. Most detected spots (spots 18–21) correspond to putative pi-class GST subunits (Q5BTY4) with different pI, but similar molecular masses between spots 18–19 (30.5 kDa) and 20–21 (29.5 kDa). Spot 23 was identified as the putative sigma1-class GST subunit (G9HSP2). In the case of *M. galloprovincialis*, six of the 11 spots were identified as cGSTs subunits, with molecular masses between 27.6 and 30.9 kDa and pI values between 5.8 and 6.7. As for *C. fluminea*, most of the detected spots (33; 34; 37; 39) are putative pi1-class GST subunits (Q8MUC3), though with different molecular masses and pI values. Spots 31 and 32 were identified as putative sigma3-class GST subunits (J71B22) with the same apparent molecular mass, but different pI. Non-GST proteins eluted from the glutathione-agarose affinity matrix were also identified for the three bivalves. In *A. cygnea*, spot 12 was identified as an E3 ubiquitin-protein ligase (K1QX18). This protein participates in the covalent conjugation of ubiquitin to protein substrates (ubiquination) for lysosomal or proteasomal degradation [31]. Spot 26 was identified in *C. fluminea* as a disintegrin and metalloproteinase with thrombospondin motifs 16 (ADAMTS-16) (K1QDA3). These enzymes are a family of 19 metalloproteases, which play an important role in the turnover of extracellular matrix proteins in various tissues [32]. Both E3 ubiquitin-protein ligase and ADAMTS-16 were co-purified, probably due to the binding through specific or non-specific interactions with the matrix or through specific protein-protein interaction with bound GST proteins.

The focus of the few studies on bivalves GSTs has been so far on the isolation and characterization of individual types of GSTs. In particular, studies on *C. fluminea* cGSTs showed that visceral mass affinity-purified extracts revealed four subunits with apparent MW between 27.2 and 30.2 kDa by SDS-PAGE analysis [18]. Analysis by non-denaturing PAGE of the same extracts revealed three acidic dimeric proteins with apparent MW of 64, 55 and 45 kDa [18]. In the same way, in this study, affinity-purified extracts from whole animals yielded four subunits with apparent MW between 29.5 and 30.5 kDa, all identified as putative pi-class subunits. According to Vidal *et al.* [18], immunoblot analysis on RP-HPLC fractions of affinity purified extracts from visceral mass and gills revealed that all subunits were related to the pi-class GSTs, with minor visceral mass subunits slightly related to mu-class ones. Most of the identified subunits in this study also belong to pi-class GSTs, and for the first time in *C. fluminea*, a sigma-class subunit was identified. In *M. galloprovincialis*, Fitzpatrick and Sheehan [12] reported that the GSH-agarose affinity-purified extract of the digestive gland contains four main proteins separable by Mono Q ion exchange chromatography with *M*_W_s ranging from 24.5 up to 27.3 kDa by SDS-PAGE. Although not characterized, GSTs from alpha, pi and sigma classes are reported in GenBank (Table 1). In this study, affinity-purified extracts from *M. galloprovincialis* yielded four pi-class and two sigma-class subunits with apparent MW between 27.6 and 30.9 kDa. For *A. cygnea*, our results constitute apparently the first GST subunits identified from these freshwater mussels. According to GenBank (Table 1), pi-class GSTs are common to other unionids, like *Hyriopsis schlegelii*, *Unio tumidus* or *Cristaria plicata*. In contrast, this is the first record of identified mu-class GSTs of the Unionidae family. Previous studies suggest that the majority of GSTs expressed in bivalve mollusks belong to the pi class and a small share to alpha, mu and sigma classes [13,18,19,21,24]. In our study, among all identified GSTs, pi-class subunits are the only ones commonly expressed in the three tested bivalves. In fact, the tendency of the pi-class majority seems to be supported by the results obtained for both *C. fluminea* and *M. galloprovincialis*. However, it is important to notice the differences found between the three species concerning pi-class isoelectric points (pI). The properties, such as pI, play a role in protein functioning. *C. fluminea* presents a more acidic cluster of pi-class subunits (pI 4.8–5.1) when compared to the found clusters in *M. galloprovincialis* and *A. cygnea*. These variations might reflect the ecological niche of each organism and constitute the molecular signatures of each bivalve.

### 2.2. cGST Activities and Kinetics

**Figure 2.**
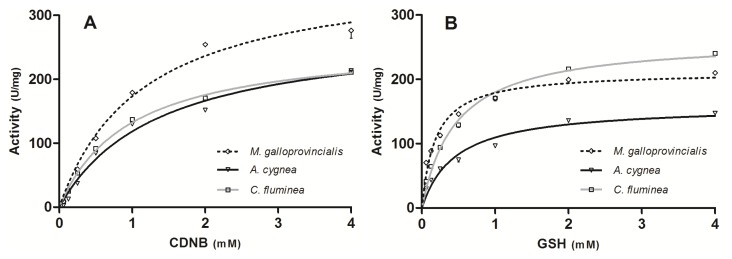
Kinetic analysis of the effects of varying GSH and CDNB concentrations on the initial rates of cGST-CDNB conjugation in *A. cygnea*, *C. fluminea* and *M. galloprovincialis*. (**A**) Varying concentrations of the electrophile CDNB from 0.0625 to 4 mM with a fixed nucleophile (GSH) concentration of 1 mM; (**B**) varying concentrations of the nucleophile GSH from 0.0625 to 4 mM with a fixed electrophile (CDNB) concentration of 1 mM. Data is expressed as the mean of *n* = 3 for all species. The error bars indicate the standard error.

**Figure 3.**
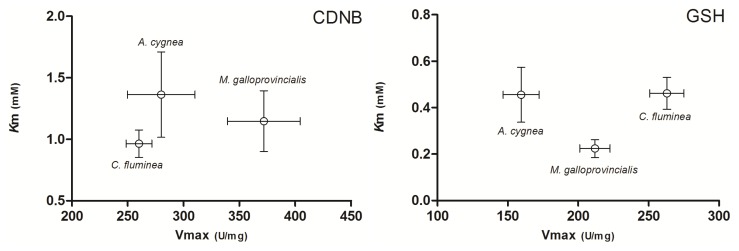
Comparison between kinetic constant values (*K*_m_ and *V*_max_) for the purified cGSTs from *A. cygnea*, *C. fluminea* and *M. galloprovincialis* using CDNB and GSH. Data is expressed as the mean of *n* = 3 for all species. The error bars indicate the 95% confidence interval.

To understand enzyme functioning, we need a kinetic description of their activity. In our work, kinetic analysis was used in order to highlight possible differences among the purified GST extracts of the three bivalve species. The purified extracts for each of the three mollusks contain a mixture of the cGST isoenzymes of the whole body of the respective bivalve. All three species of bivalves demonstrated classic Michaelis–Menten enzyme saturation curves for both varying concentrations of 1-chloro2,4-dinitrobenzene (CDNB) and GSH (Figure 2). Enzyme saturation curves fitted the Michaelis–Menten model with regression coefficients of 0.954/0.980 (GSH/CDNB) for *A. cygnea*, 0.985/0.994 for *C. fluminea* and 0.954/0.984 for *M. galloprovincialis*. The comparison between apparent *K*_m_ and *V*_max_ values derived from these plots are shown in Figure 3. For the catalytic activity of purified cGSTs toward CDNB, the apparent *K*_m_ values present no significant differences among the three species (*p* < 0.05), ranging between 0.964 and 1.363 mM. Regarding *V*_max_, the value for *M. galloprovincialis* (371.9 ± 32.5 units per milligram (U/mg)) is significantly higher compared to *A. cygnea* (279.9 ± 30.3 U/mg) and *C. fluminea* (260.1 ± 11.5 U/mg) (*p* < 0.05). For the catalytic activity of cGSTs toward GSH, *K*_m_ value for *M. galloprovincialis* (0.224 ± 0.03 mM) is significantly lower compared to *A. cygnea* (0.455 ± 0.11 mM) and *C. fluminea* (0.461 ± 0.06) (*p* < 0.05). *V*_max_ values for *A. cygnea* (159.5 ± 12.8 U/mg), *M. galloprovincialis* (211.9 ± 10.7 U/mg) and *C. fluminea* (263.0 ± 12.2 U/mg) are significantly different among the three species of bivalves (*p* < 0.05).

Interestingly, the three species evidence different enzymatic behavior for both CDNB and GSH. As for Vidal and Narbonne [17], the resulting *K*_m_ values for CDNB are higher for all species when compared to the ones obtained for GSH, indicating a higher affinity of purified cGST for GSH than for the conjugating substrate. Regarding CDNB, the three species show similar affinities for the substrate. Still, *M. galloprovincialis* presents a significantly higher *V*_max_ compared to *A. cygnea* and *C. fluminea*. For GSH, all *V*_max_ values are statistically different, following the order *C. fluminea* > *M. galloprovincialis* > *A. cygnea*. The marine mussel, *M. galloprovincialis*, shows a significant higher affinity for GSH towards the other two mollusks. We should point out that the differences in *V*_max_ values obtained among the three bivalve species can be conditioned by the varying amounts of non-GST proteins between the different preparations, since the pool of purified cGST also contains few additional non-GST proteins.

## 3. Experimental Section

### 3.1. Test Species and Cultures

All bivalves were collected in the summer of 2012. *C. fluminea* (25–30 mm shell length) was manually collected in the estuary of Minho River (Valença, Portugal); *A. cygnea* (100–105 mm shell length) was collected from Lake Mira (Aveiro, Portugal); *M. galloprovincialis* (65–70 mm shell length) was collected from Memória Beach (Porto, Portugal). These areas have been used as reference sites in the case of previous studies [33,34]. The bivalves were acclimatized in the laboratory for at least fourteen days at 18 ± 1 °C and kept in tanks with aerated tap water, under a light:dark regime of 16:8 h. The animals were fed with an algal suspension (*Chlorella vulgaris*, 1 × 10^5^ cells mL^−1^) twice a week. *C. vulgaris* (LEGE Z-001) was obtained from the Culture Collection of LEGE (CIIMAR, Porto, Portugal). This Chlorophycea was cultured in 6-L flasks containing 4 L of Z8 medium [35] using cool white fluorescent light (10 μmol m^−2^ s^−1^) under a light:dark regime of 14–10 h and a temperature of 25 ± 1 °C.

### 3.2. cGST Purification

#### 3.2.1. Enzyme Extraction

Enzyme extracts were prepared at 4 °C, using a similar method as described in Vasconcelos *et al.* [36]. Bivalves (minimum wet weight, 10 g; at least 3 individuals; whole body) were frozen in liquid nitrogen and ground to a fine powder. Several extractions per bivalve tissue were done. For each, 2 g of ground tissue were homogenized on ice in 10 mL of sodium phosphate buffer (0.1 M, pH 6.5) containing 20% glycerol (*v*/*v*), 1.4 mM dithioerythritol (DTE), 1 mM ethylenediaminetetraacetic acid (EDTA). Cell debris was removed by centrifugation at 4600× *g* for 10 min (4 °C), and the supernatant was centrifuged again at 100,000× *g* for 1 h (4 °C). The remaining supernatant was precipitated between 0 and 35% (*w*/*v*) and then between 35% and 80% (*w*/*v*) with ammonium sulfate. The pellet obtained from the second precipitation was resuspended in 20 mM sodium phosphate buffer, pH 7.0, and desalted by gel filtration on P-10 Desalting Columns (Sephadex G-25 Medium, GE Healthcare, Uppsala, Sweden), according to the manufacturer’s protocol. The eluate was frozen in liquid nitrogen and kept at −70 °C until further use.

#### 3.2.2. Affinity Chromatography

Cytosolic enzyme extracts were purified on the basis of their affinity to GSH using GSH-agarose prepacked columns (Sigma-Aldrich, St. Louis, MO, USA), according to the protocol supplied by Sigma-Aldrich. Briefly, the column was equilibrated with 10 mM phosphate buffered saline (PBS) (pH 7.4; 150 mM NaCl) before applying cytosolic enzyme extract (2 mL). The column was then washed with PBS 1% Triton X-100 to remove non-specifically bound proteins. Finally, the bound GSTs were eluted with 4 mL of 10 mM GSH in 50 mM Tris-HCl (pH 8.0) buffer, and 1 mL fractions were collected and analyzed for enzyme activity and the amount of protein. Enzymatically active pooled fractions were pooled and concentrated using Vivaspin 2-mL concentrators (<10 kDa polyethersulfone membrane) (Sartorius Stedim Biotech, Goettingen, Germany) by centrifuging at 3000× *g* for 30–60 min (4 °C). Protein samples were stored at −70 °C.

### 3.3. cGST Activities and Kinetics

GST activity with CDNB was measured as described by Habig *et al.* [2], adapted to microplate, following the procedure described in Frasco and Guilhermino [37]. All assay incubations were conducted at 25 °C. CDNB was dissolved in ethanol, with a final reaction concentration of less than 0.01%, and GSH was dissolved in buffer. For all assays, the reaction mixture (300 μL final volume) contained 2.5 μg of purified cytosolic protein, along with substrate, GSH and sodium phosphate buffer (0.1 M) with pH 7.2. Preliminary studies using 0.1 M sodium buffers prepared over a range of pH values (6.5, 6.8, 7.2 and 7.6) were conducted in order to determine the optimal pH value. Enzyme activity was calculated in units (U), which is the amount of enzyme that catalyzes the transformation of 1 micromole of substrate per minute and is given in units per milligram (U/mg) of protein. Protein quantification was conducted using a microplate adapted protocol of the Bradford method [38] and the FluoroProfile quantification kit (Sigma-Aldrich, St. Louis, MO, USA). All determinations were conducted in triplicate on a temperature-controlled Bioteck microplate reader (Synergy HT, 2009, BioTek, Winooski, VT, USA) in 96-well microplates. The Michaelis constant (*K*_m_) and maximum velocity (*V*_max_) determinations with CDNB as a substrate for cGST assays from the three bivalves were performed in triplicate with varying concentrations of CDNB (0.0625–4 mM) and a constant GSH concentration (1 mM). The *K*_m_ and *V*_max_ with GSH as a substrate were performed with varying concentrations of GSH (0.0625–4 mM) and a constant CDNB concentration (1 mM). The kinetic parameters were determined using non-linear regression in Graphpad Prism (Graphpad Software, San Diego, CA, USA).

### 3.4. Two-Dimensional Gel Electrophoresis

#### 3.4.1. Isoelectric Focusing, SDS-PAGE and Image Acquisition

Each protein sample was dissolved in rehydration buffer consisting of urea (7 M), thiourea (2 M), 3-[(3-cholamidopropyl) dimethylammonio]-1-propane sulfonate (CHAPS) (4%, *w*/*v*), dithiothreitol (65 mM) and ampholytes, pH 4–7 (0.8%, *v*/*v*) (Bio-Rad, Hercules, CA, USA), as described in Campos *et al.* [39]. A total of 125 μL of this solution, containing 15 μg protein, were used to rehydrate each 7 cm, pH 4–7, ReadyStrip IPG strip (Bio-Rad, Hercules, CA, USA). Two IPG strips were used per sample. First-dimension, isoelectric focusing was then performed at 20 °C using the following single step program: 0–4000 V, rapid voltage ramp, 10,000 V h^−1^ [39]. Prior to the second-dimension electrophoresis, each IPG strip was equilibrated in 2.5 mL of 6 M urea, 2% SDS, 0.1 mM EDTA, 50 mM Tris, pH 6.8, 30% glycerol, 0.002% bromophenol blue and 65 mM DTT for 15 min, followed by 15 min with 260 mM iodoacetamide substituting DTT. Each strip was then placed on top of a 1 mm-thick, 10% acrylamide SDS-PAGE gel. Molecular weight markers were loaded onto filter paper and placed next to the IPG strip. Low-melting point agarose was used to cover the IPG strip and filter paper. Two gels were run simultaneously in a Mini Protean Tetra cell (Bio-Rad, Hercules, CA, USA), at 200 V for approximately 45 min. The gels were stained using Coomassie G-250 stain (SimplyBlue, Invitrogen, Carlsbad, CA, USA), according to the manufacturer’s instructions. Gel images (400 dpi) were obtained using a GS-800 Calibrated Densitometer (Bio-Rad, Hercules, CA, USA) and analyzed using the PDQuest 2-D Analysis Software (Bio-Rad, Hercules, CA, USA). All spots were selected for peptide mass fingerprinting analysis.

#### 3.4.2. Protein Identification

Spots of interest were manually excised from the gels, placed onto 96-well microplates and stored at −20 °C until digestion. Gel plugs were destained by a first wash with ultra-pure water and two additional washes with 50% acetonitrile (ACN). The gel plugs were dried with 100% ACN and were then rehydrated with a solution of trypsin (sequencing grade; Promega, Madison, WI, USA) (6.7 mg mL^−1^) [39]. Digestion occurred overnight at 37 °C. Protein digests were desalted and concentrated using ZipTips (Millipore, Bedford, MA, USA), following the manufacturer’s instructions. Samples were crystallized onto a stainless steel 192-well MALDI plate using the dried droplet method. For the matrix, a solution of 5–10 mg mL^−1^ α-cyano-4-hydroxycinnamic acid in 50% ACN/0.1% trifluoroacetic acid was used. Samples were analyzed using the 4700 Proteomics Analyzer MALDI-TOF/TOF (Applied Biosystems, Foster City, CA, USA). Peptide mass fingerprint data were collected in positive MS reflector mode in the range of 700–4000 mass-to-charge ratio (*m*/*z*) using 1000–3000 laser shots for each sample and calibrated internally using trypsin autolysis peaks. Several of the highest intensity nontrypsin peaks were selected for tandem MS (MS/MS) analysis. The spectra were analyzed using GPS Explorer (Version 3.6; Applied Biosystems, Foster City, CA, USA), which acts as an interface between the Oracle database containing raw spectra and a local copy of the Mascot search engine (Version 2.1.04, Matrix Science, London, UK). The MS and MS/MS data were searched together against a locally stored copy of the NCBInr and SwissProt using the Mascot search engine. The search included peaks with a signal-to-noise ratio greater than 10 and allowed for up to two missed trypsin cleavage sites. To be considered a match, a confidence interval (CI) calculated by Applied Biosystems GPS Explorer (GPS), of at least 95%, was required.

## 4. Conclusions

In summary, the present study provides for the first time the cGST proteome collection in three bivalve species. Using proteomics, we were able to identify in the affinity-purified extracts from *M. galloprovincialis*, *C. fluminea* and *A. cygnea* several GST subunits. For *A. cygnea*, the identified cGSTs subunits in this study constitute the first GST record for these freshwater mussels. The differences found in cGSTs collection (diversity and properties) and kinetics might justify diverse selective advantages for each bivalve organism. Therefore, future studies should focus on investigating the more efficient biotransformation GST isoforms in the physiological response of bivalves to xenobiotics exposure.

## Supplementary Information



## Figures and Tables

**Table 1.** Distribution of cytosolic glutathione transferase (cGST) isoforms that are present in bivalve species (sequences obtained from GenBank; details in Table S1).

Species	Mu	Pi	Alpha	Sigma	Theta	Zeta	Omega	Rho
*Chlamys farreri*		×				×		
*Corbicula fluminea*		×						
*Crassostrea ariakensis*	×			×			×	
*Crassostrea gigas*	×			×			×	
*Cristaria plicata*		×						
*Dreissena polymorpha*		×						
*Hyriopsis schlegelii*		×						
*Laternula elliptica*		×						×
*Mercenaria mercenaria*		× (2)						
*Mytilus edulis*		×						
*Mytilus galloprovincialis*		×	×	× (3)				
*Ostrea edulis*				×			×	
*Pinctada fucata*							×	
*Ruditapes philippinarum*	×	×		× (3)	×		×	×
*Saccostrea palmula*	×							
*Solen grandis*				×				
*Unio tumidus*		×						

( ), the number of isoforms.

**Table 2.** Identification of the affinity chromatography purified proteins from *A. cygnea*, *C. fluminea* and *M. galloprovincialis*.

Spot	Observed PI/MR (10^3^)	Theoretical PI/MR (10^3^)	Putative Identification	CI (%)	Homology to Protein	Molecular Activity
*Anodonta cygnea*						

2	4.7/26.2	12.1/7.2	Uncharacterized protein	99.8	H2XZV7 *Ciona intestinalis*	-
4	4.9/22.3	12.1/7.2	Uncharacterized protein	99.9	H2XZV7 *Ciona intestinalis*	-
7	5.5/28.0	8.3/24.6	GST mu-class	99.9	B3VDE4 *Haliotis discus*	Transferase
8	5.7/27.8	8.6/25.1	GST mu-class	99.9	A7LFK1 *Cyphoma gibbosum*	Transferase
12	6.4/28.7	5.1/163.3	E3 ubiquitin-protein ligase CHFR	95.8	K1QX18 *Crassostrea gigas*	Ligase
15	6.7/27.4	7.6/23.4	GST pi	100	E1B2Z8 *Cristaria plicata*	Transferase

*Corbicula fluminea*						

17	4.8/36.5	12.1/7.2	Uncharacterized protein	97.2	H2XZV7 *Ciona intestinalis*	-
18	4.8/30.5	4.9/23.7	GST pi-class	99.9	Q5BTY4 *Corbicula fluminea*	Transferase
19	5.0/30.5	4.9/23.7	GST pi-class	100	Q5BTY4 *Corbicula fluminea*	Transferase
20	5.0/29.5	4.9/23.7	GST pi-class	100	Q5BTY4 *Corbicula fluminea*	Transferase
21	5.1/29.5	4.9/23.7	GST pi-class	100	Q5BTY4 *Corbicula fluminea*	Transferase
23	5.6/28.2	6.1/23.3	GST sigma1-class	95.4	G9HSP2 *Venerupis philippinarum*	Transferase
26	6.1/30.3	8.8/114.6	ADAMTS-16	98.0	K1QDA3 *Crassostrea gigas*	Metalloprotease

*Mytilus galloprovincialis*						

31	5.8/28.7	5.4/22.9	GST sigma3-class	100	J7IB22 *Mytilus galloprovincialis*	Transferase
32	5.8/27.6	5.4/22.9	GST sigma3-class	100	J7IB22 *Mytilus galloprovincialis*	Transferase
33	6.0/29.8	5.9/23.8	GST pi1-class	100	Q8MUC3 *Mytilus galloprovincialis*	Transferase
34	6.3/30.9	5.9/23.8	GST pi1-class	100	Q8MUC3 *Mytilus galloprovincialis*	Transferase
37	6.5/28.4	5.9/23.8	GST pi1-class	100	Q8MUC3 *Mytilus galloprovincialis*	Transferase
39	6.7/29.0	5.9/23.8	GST pi1-class	100	Q8MUC3 *Mytilus galloprovincialis*	Transferase
