# Supplementary material for: Proteomic Profiling of Cytosolic Glutathione Transferases from Three Bivalve Species: Corbicula fluminea, Mytilus galloprovincialis and Anodonta cygnea
Source: Int J Mol Sci. 2014 Jan 27;15(2):1887–900. doi: 10.3390/ijms15021887 (PMC3958827; doi:10.3390/ijms15021887)
Supplement: Supplementary file 1 [file ijms-15-01887-s001.pdf]

Supplementary Information

Table S1. Bivalve GST sequences in GenBank.

Species name	Class	Isoform	GI
Chlamys farreri	zeta	-	GU361617.1
Chlamys farreri	pi	-	FJ588638.4
Corbicula fluminea	pi	-	AY885667.1
Crassostrea ariakensis	omega	-	EU908273.1
Crassostrea ariakensis	mu	-	EU908274.1
Crassostrea ariakensis	sigma	-	EU908270.1
Crassostrea gigas	omega	-	AJ557141.1
Crassostrea gigas	mu	-	AJ558252.1
Crassostrea gigas	sigma	-	AJ577235.1
Cristaria plicata	pi	-	HQ166721.1
Dreissena polymorpha	pi	-	EF194203.1
Hyriopsis schlegelii	pi	-	EU145724.1
Laternula elliptica	rho	-	FJ615307.1
Laternula elliptica	pi	-	EU131183.1
Mercenaria mercenaria	pi	2	EU024654.1
Mercenaria mercenaria	pi	1	EU024656.1
Mytilus edulis	pi	-	AY557404.1
Mytilus galloprovincialis	pi	1	AF527010.1
Mytilus galloprovincialis	sigma	1	JX485636.1
Mytilus galloprovincialis	sigma	2	JX485637.1
Mytilus galloprovincialis	sigma	3	JX485638.1
Mytilus galloprovincialis	alpha	-	JX485635.1
Ostrea edulis	omega	-	JN091800.1
Ostrea edulis	sigma	-	JN091840.1
Pinctada fucata	omega	-	GU3625421
Ruditapes philippinarum	omega	-	HM061130.1
Ruditapes philippinarum	rho	-	JN388954.1
Ruditapes philippinarum	theta	-	JF499392.1
Ruditapes philippinarum	mu	-	JN593116.1
Ruditapes philippinarum	pi	-	FJ516741.2
Ruditapes philippinarum	sigma	1	JN388948.1
Ruditapes philippinarum	sigma	2	JN388949.1
Ruditapes philippinarum	sigma	3	JN388950.1
Saccostrea philippinarum	mu	-	FJ527304.1
Solen grandis	sigma	-	JN642123.1
Unio tumidus	pi	-	AY885666.1

© 2014 by the authors; licensee MDPI, Basel, Switzerland. This article is an open access article distributed under the terms and conditions of the Creative Commons Attribution license (http://creativecommons.org/licenses/by/3.0/).
